# A Versatile Microchannel Array Device for Portable and Parallel Droplet Generation

**DOI:** 10.1002/smsc.202400005

**Published:** 2024-03-21

**Authors:** Zhengmin Tang, David Eun Reynolds, Caishu Lv, Dandan Zhang, Jina Ko, Yongcheng Wang

**Affiliations:** ^1^ Department of Laboratory Medicine of The First Affiliated Hospital & Liangzhu Laboratory Zhejiang University School of Medicine Hangzhou 311121 China; ^2^ Department of Bioengineering Department of Pathology and Laboratory Medicine University of Pennsylvania Philadelphia 2926 USA; ^3^ Department of engineering Xinhaohui Biotech Hangzhou 311121 China; ^4^ Department of Pathology Key Laboratory of Disease Proteomics of Zhejiang Province School of Medicine Zhejiang University Hangzhou 310058 China

**Keywords:** digital polymerase chain reaction, high‐throughput generation, microfluidics, monodispersed droplets

## Abstract

The efficient generation of monodispersed droplets holds great promise for micro‐/nanoparticle synthesis and biochemical analysis. However, it remains a challenge to achieve high‐throughput generation of monodispersed droplets in a portable and/or parallel manner in nonexpert biomedical laboratories. Herein, a versatile microchannel array (μCA) device is reported that is portable, multifaceted, reliable, and mass‐manufacturable, supporting the high‐throughput generation of monodispersed droplets in different ways. This device consists of a silicon‐based μCA chip based on the step emulsification principle, as well as two matching plastic containers, both of which can be mass‐manufactured by traditional microfabrication methods at low material costs. With the μCA device, aqueous solution can be dispersed into emulsion droplets by various modes, such as mechanical pump‐based large‐scale, handheld syringe‐based portable, and gas pump‐based highly parallel droplet generation. Furthermore, it is demonstrated that our cost‐effective device can be applied for digital polymerase chain reaction analysis, supporting the need for more accessible microfluidic systems. Thus, the present device is expected to have a significant impact on both benchside and bedside applications.

## Graphical abstract

A versatile microchannel array device that is portable, multifaceted, reliable, and mass‐manufacturable, supports the high‐throughput generation of monodispersed droplets in different ways, which provides a versatile droplet platform for a myriad of different applications.

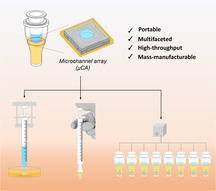

## 1 Introduction

Monodispersed water‐in‐oil (W/O) emulsion droplets have served as versatile platforms for polymeric particle synthesis^[^ ^1^, ^2^, ^3^, ^4^ ^]^ and microreactors for biochemical analysis.^[^ ^5^, ^6^, ^7^ ^]^ The common approach for producing these free‐standing droplets involves droplet microfluidics. Droplet microfluidics manipulates discrete droplets through immiscible multiphase flows inside microchannels, generating uniform droplets with controlled size and chemical composition.^[^ ^8^ ^]^ The most frequently used droplet‐based microfluidics is based on “T‐junction”^[^ ^9^ ^]^ and “flow focusing”^[^ ^10^ ^]^ designs, which commonly require sophisticated microfluidic chips and exquisite control over the flow rates. Therefore, it remains a challenge to achieve high‐throughput generation of monodispersed droplets in a portable and/or parallel way, especially in nonexpert biomedical laboratories.

Step emulsification is a low‐shear method that spontaneously breaks up dispersed fluids under the geometry‐induced Laplace pressure difference.^[^ ^11^, ^12^ ^]^ The monodispersity of generated droplets is insensitivity to flow rates, which allows for the incorporation of common pressure to drive the dispersed phase into emulsion droplets. Pioneered by Sugimura et al.^[^ ^13^ ^]^ a considerable body of research dedicated to exploring the design, mechanism, and application of step emulsification devices.^[^ ^14^, ^15^, ^16^ ^]^ However, the implementation of most step emulsification devices involves connection tubing and/or multiple pumps, which could limit the portability and hinder the possibility of parallel droplet generation for multiple samples. Recently, many efforts have been devoted to developing chip‐free emulsion techniques to bypass traditional microfluidic devices. These systems include cross‐interface emulsification,^[^ ^17^ ^]^ droplet printing,^[^ ^18^ ^]^ membrane emulsification,^[^ ^19^ ^]^ pipette‐tips/capillary‐based approaches,^[^ ^20^, ^21^ ^]^ and centrifugal emulsification.^[^ ^22^, ^23^, ^24^ ^]^ Together, advances in these droplet‐based methods have promoted a revolution in digital analysis of biomolecules.^[^ ^21^, ^23^, ^25^, ^26^, ^27^ ^]^ Nevertheless, the mass manufacturing ability and reliability of these newly developed methods require further investigations.

Here, we report a versatile microchannel array (μCA) device that is portable, multifaceted, reliable, and mass‐manufacturable, supporting the high‐throughput generation of monodispersed droplets in different ways. This μCA device was demonstrated to possess multiple droplet generation modes to meet various application needs, including mechanical pump‐based large‐scale, handheld syringe‐based portable, and gas pump‐based highly parallel droplet generation. Finally, the application of μCA device for digital polymerase chain reaction (dPCR) was also demonstrated.

## 2 Results

### 2.1 Design of the μCA Device

**Figure 1:**
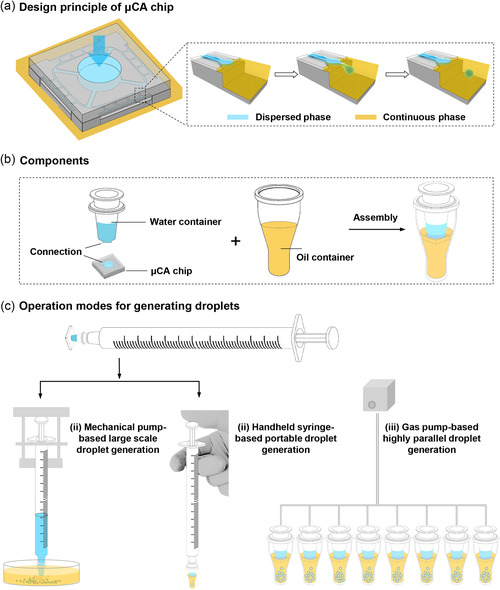
Design principle, components, and operation modes of the μCA device. a) 3D image of the μCA chip. The general design of the μCA is based on the step emulsification. Droplet formation is facilitated by the sudden expansion at the nozzles. b) Designed components of the μCA, including water container and oil container. c) Operation modes for generating droplets. The assembled device can be equipped with a single syringe or fitted with a gas pump. The μCA chip connected with water container was immersed into the oil before droplet generation. The droplets can be generated through different operation modes: i) mechanical pump‐based large‐scale droplet generation, ii) handheld syringe‐based portable droplet generation, and iii) gas pump‐based highly parallel droplet generation.

The architecture of the μCA chip is illustrated in **Figure** **1**a. The general design of the μCA is based on the step emulsification principle, which features low shear, insensitivity to flow rate, and a simple structure that is easy to integrate. In particular, the main body of the μCA chip consists of two layers, the lower layer is a silicon substrate with a photolithography microchannel array, and the upper layer is a bonded glass with a cylindrical through‐hole. The silicon substrate was etched with four identical grooved‐type nozzles and a terrace on each side, and there was a deeply etched well over the terrace end. When a common pressure is applied toward the through hole of the glass, the water (dispersed phase) passes through the microchannel, and the droplets generate continuously at the nozzles. The droplets then move into the oil (continuous phase) to form stable emulsion.

Several components were implemented into the μCA device to make it an economically sound product. As shown in Figure 1b, we designed a water container with a protruding bottom whose dimensions matched to the through‐hole of the bonding glass. Thus, the water container can be connected with the μCA chip, and the solution does not leak out from the junction. For droplet collection, we designed a cup‐shaped oil container that has dimensions attuned to a 96‐well plate so that up to 96 × *N* samples can be emulsified simultaneously. Unlike other microfluidic systems, droplet generation using the μCA device does not need connection tubing and exquisite control of flow rates by external forces. This novel design of device makes it possible to disperse aqueous solution into emulsion droplets by exploiting various droplet generation modes, including mechanical pump‐based large‐scale, handheld syringe‐based portable, and air pump‐based highly parallel droplet generation (Figure 1c). Therefore, this μCA device is versatile for a myriad of applications, such as large‐scale synthesis of polymeric microparticles, point‐of‐care diagnosis, and parallel digital single‐molecule analysis.

### 2.2 Fabrication of the μCA Device

**Figure 2:**
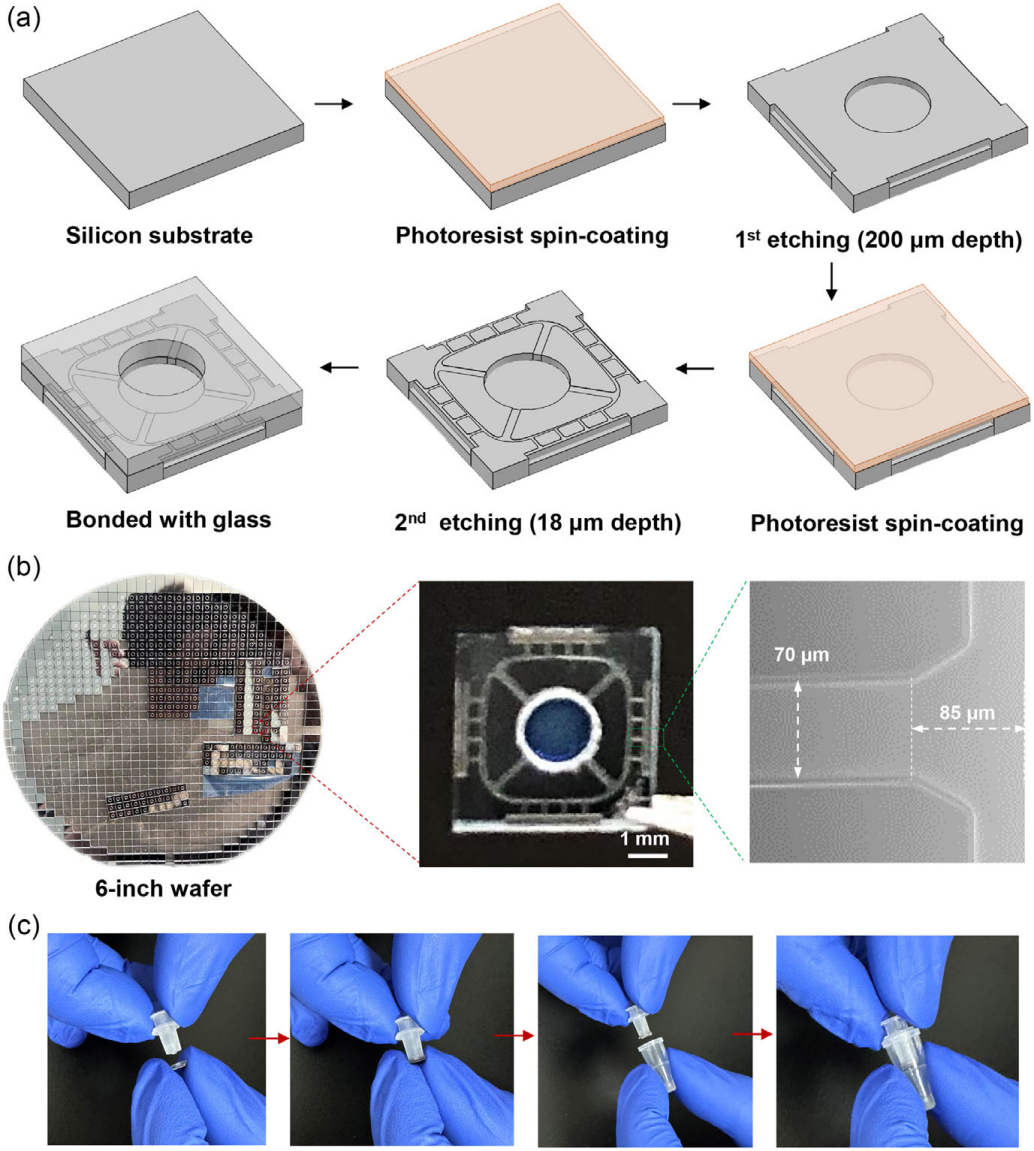
Fabrication, characterization, and assambly of μCA device. a) Schematic of a two‐step photolithography procedure employed in the fabrication of μCA. b) A 6‐inch silicon wafer containing over 800 μCA chips. The enlarged micrographs of the μCA show the geometry and the sizes of microchannels and nozzles. c) Photos showing that the μCA chip and the containers can be easily assembled into a device.

The μCA chip can be readily mass‐manufactured by traditional microfabrication techniques. **Figure** **2**a depicts the schematic of a two‐step photolithography procedure employed in the fabrication of μCA. Initially, a 200 μm depth etching was utilized to create the circular main channel and four surrounding wells on a square silicon substrate. Subsequently, in the second lithography step, tributary channels, terrace, and parallel nozzles were etched to a depth of 18 μm. Following the etching process, the silicon substrate was permanently bonded to a through‐holed glass through anodic bonding to seal the microchannels. In practice, over 800 μCA chips were simultaneously constructed on a 6 inch silicon wafer, and laser cutting was employed to make individual μCA chips (Figure 2b). In general, the diameter of the generated droplets can be tuned by tailoring the μCA chip with varied nozzle depth, width, or terrace length. In this work, we designed the following parameters of the μCA chip as an example to produce high‐quality W/O emulsion droplets: nozzle width ≈70 μm, nozzle depth ≈18 μm, and terrace length ≈85 μm, respectively. In addition to the μCA chip fabrication, the plastic water and oil containers can be massively manufactured through injection molding. The μCA chip and corresponding components can be easily assembled without the need for connection tubing (Figure 2c). Furthermore, the average material cost for the fabrication of μCA device was estimated to be USD <$0.5 (Table S1, Supporting Information), which is comparable to the cost for routine plastic consumables in biomedical laboratories. Compared with other microfluidic systems,^[^ ^8^ ^]^ our fabrication approach for the μCA device has been demonstrated to be more reliable and can achieve large‐scale production at relatively low costs, which will allow the μCA device to be adopted by other biomedical laboratories for droplet‐based research.

### 2.3 Multiple Operation Modes for Stable Droplet Generation

**Figure 3:**
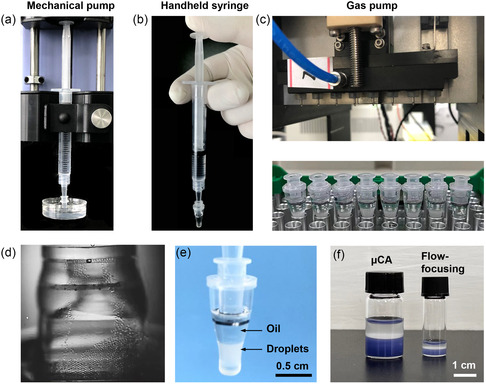
Operation modes of the μCA device. a–c) Images showing multiple operation modes for stable W/O droplet generation: a) mechanical pump‐based large‐scale droplet generation, b) handheld syringe‐based portable droplet generation (see Video S2), and c) gas pump‐based highly parallel droplet generation. The emulsion droplet (oil) container can be fitted to a commercial PCR instrument without droplet transfer for digital analysis applications. d) Video snapshot showing the droplet generation process (see Video S1). e) The W/O emulsions stably sit at the bottom of the oil container. f) Visual comparison of the droplet generation rate of the μCA device versus a traditional flow‐focusing device. Trypan blue dye was added to aqueous solution before droplet generation for visualization.

With the μCA device, monodispersed droplets can be produced by multiple operation modes: 1) mechanical pump‐based droplet generation, which could generate large‐scale monodispersed emulsion droplets (**Figure** **3**a); 2) handheld syringe‐based droplet generation, which enables portable, rapid, and stable droplet generation for nonexpert biomedical laboratories (Figure 3b); and 3) gas pump‐based highly parallel droplet generation, as the gas pump could provide a common pressure to generate emulsion droplets for multiple samples simultaneously (Figure 3c). To illustrate how the droplets were formed with the μCA device, a high‐speed camera was used to monitor the detailed droplet generation process. Figure 3d and Video S1 show that monodispersed droplets were continuously formed on the nozzles and migrated into the oil. The droplet emulsion was stably sited at the bottom of the container as the light mineral oil was used as a continuous phase (Figure 3e). The droplet generation rate enabled by the μCA device demonstrated the capacity to produce ten times the number of droplets compared to the traditional flow‐focusing devices (Figure 3f). Notably, more parallel microchannels and nozzles can be incorporated into the device to increase the droplet production rate, supporting its potential for the large‐scale industrial production of monodispersed emulsion droplets.

**Figure 4:**
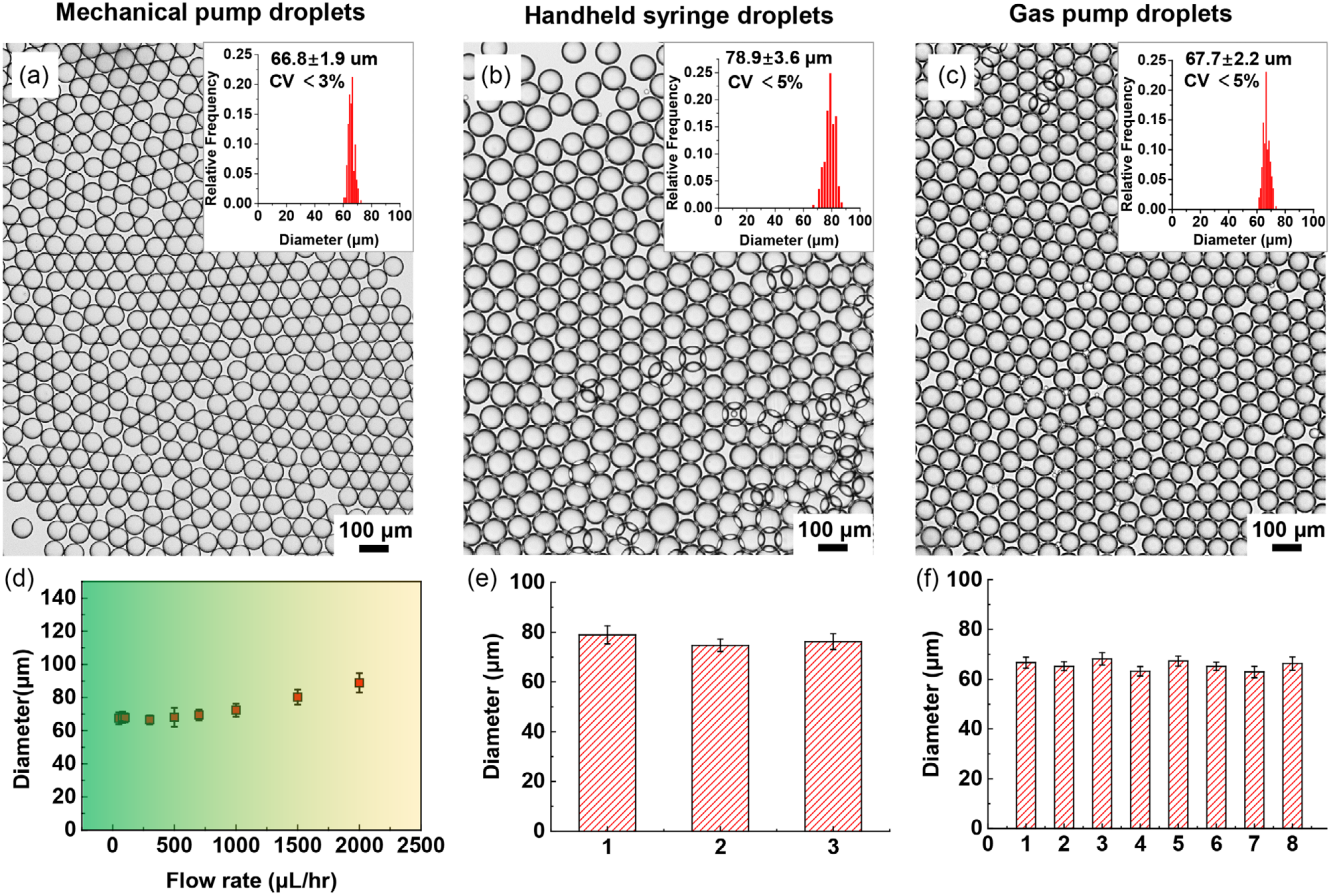
Multiple operation modes for stable droplet generation. a–c) Micrographs of the monodispersed W/O droplet generated by a) mechanical pump‐based large‐scale droplet generation, b) handheld syringe‐based quick droplet generation, and c) gas pump‐based highly parallel droplet generation. Insets show the diameter distributions of the droplets. d) The diameter of droplets generated at different flow rates by mechanical pump‐based droplet generation, with error bars representing the standard deviation of droplet diameter. Droplet diameters were examined across 300 droplets. e, f) The measured diameter and standard deviation of the droplets generated by e) handheld syringe with 3 repeated experiments and f) gas pump for 8 parallel samples.

The size and uniformity of droplets were assessed regarding different droplet generation modes as described previously (**Figure** **4**). The quality of generated droplets enabled by the mechanical pump was initially characterized. Overall, the droplets have a diameter of ≈67 μm with a low coefficient of variation (CV) of 2%–5% when the aqueous phase flow rate is below 1 mL h^−1^ (Figure 4a). The droplets become slightly larger than ≈70 μm while maintaining uniformity, with an elevation in flow rate to above 1 mL h^−1^ (Figure 4d and Figure S1, Supporting Information). When heavy Novec 7500 oil was used as the continuous phase, monodispersed droplets could also be generated (Figure S2, Supporting Information). Moreover, the μCA device can be operated by manual injection of the dispersed phase with a syringe to produce droplets with a diameter of ≈79 μm (CV < 5%) (Figure 4b and Figure S3, Supporting Information). Repeated experiments were performed to confirm the stability and reproducibility of the droplet production by handheld syringe injection. As shown in Figure 4e, 50 μL of monodispersed droplets with diameters of ≈79 μm (CV < 5%) were obtained from three different experimenters within 30 s. For highly parallel droplet generation, the gas pump was used to provide a common pressure for multiple μCA devices. As shown in Figure 4c,f, monodispersed droplets with diameters of ≈68 μm (CV < 5%) were obtained from 8 parallel devices. These results suggested that our μCA devices exhibit high‐throughput and acceptable stability that meet different needs for droplet‐based applications.

### 2.4 dPCR Based on μCA Device

**Figure 5:**
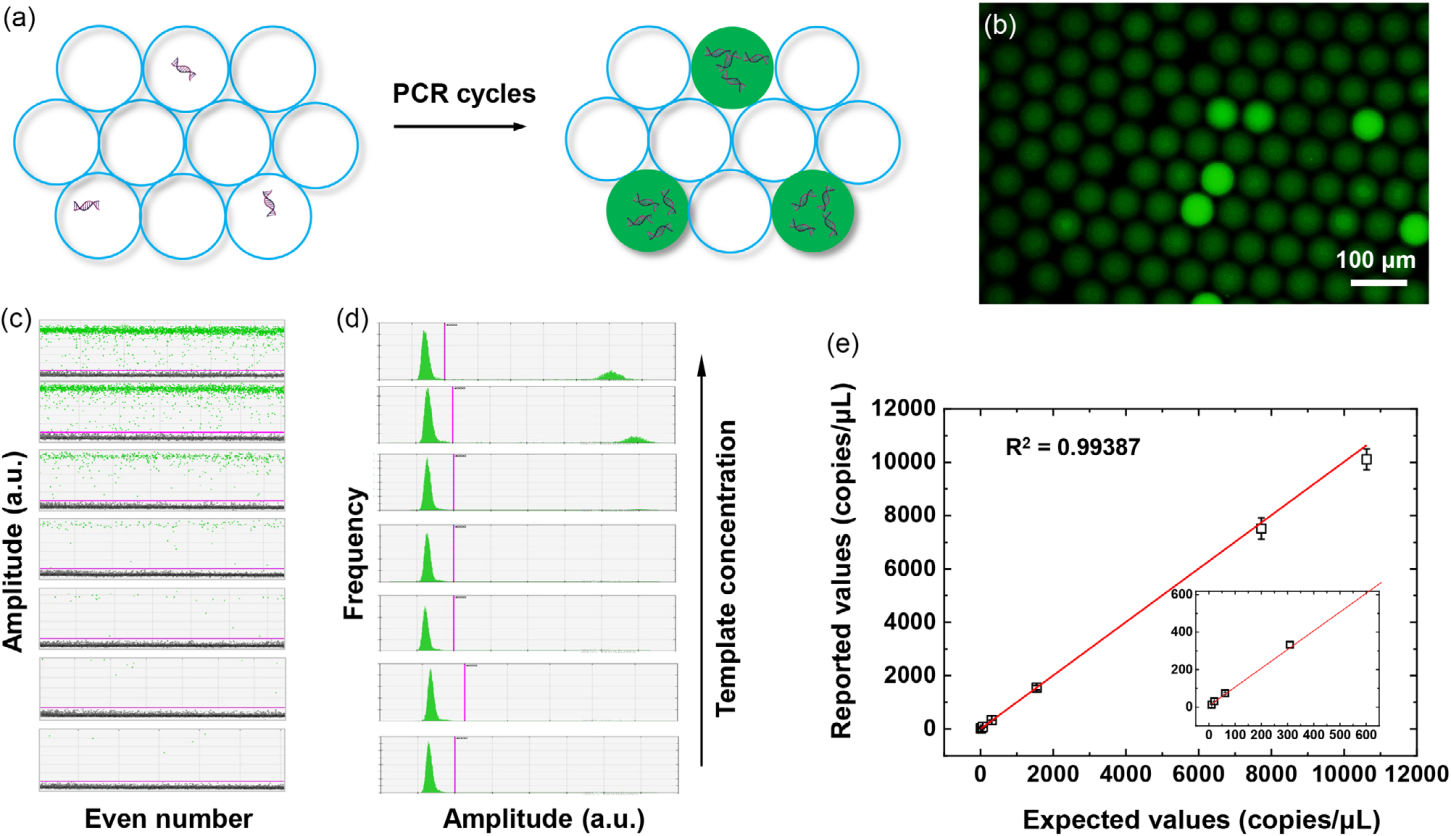
Results of digital PCR from μCA device. a) Collected droplet signals from PCR are assessed to quantify the number of nucleic acids. The droplets containing DNA copies are positive (green) after PCR. b) Fluorescence images indicating the digital amplification within the emulsion. c) Scatter plots and d) distributions of fluorescence counting results with respect to the decreased concentrations of templated DNA. The green and gray points indicate a positive and negative signal, respectively. e) The concordance between the results of DNA concentration calculated based on Poisson distribution and the theoretically expected values.

For application purposes, we applied the μCA device to produce droplets for dPCR. The gas pump was chosen as the external driving force for parallel droplet generation. As the oil container was used to collect the emulsion droplets, extra emulsion transfer steps were avoided and minimum sample loss was achieved. The template was a double‐stranded DNA fragment, whose sequence was from the EGFR T790M gene (Table S2, Supporting Information). The total aqueous volume of each dPCR was 25 μL, with 5 μL of sample and 20 μL of premix buffer containing polymerase, primers, and TaqMan probes (Table S3, Supporting Information). Upon gas pump injection, the 25 μL reaction mix completely transformed into emulsion droplets with almost zero dead volume. The droplet generation process for multiple samples took less than 3 min, resulting in highly uniform droplets with a diameter of around 70 μm. The PCR thermocycling was performed directly with the receptor oil containers on a conventional PCR machine. After PCR, the droplets were divided into “positive” and “negative” fluorescent groups that indicate the existence and absence of the template DNA, respectively (**Figure** **5**a,b). Droplet counting and fluorescence signal measurements were performed using a Bio‐Rad dPCR reader. dPCR tests were carried out for 7 samples, with serial diluted concentrations of target template DNA from a few copies/μL to ≈10 000 copies/μL. Each experiment was performed three times for scientific rigor, and the results are presented in Figure 5c–e. As the DNA template concentrations in the 7 samples were gradually decreased, the fraction of positive droplets decreased as well. The measured DNA concentration was calculated according to the Poisson distribution of the original DNA template in droplets. A linear trend was observed between the reported and expected DNA concentrations between 1 and 10 000 copies/μL (Figure 5e). Moreover, the measured DNA concentrations for the three repeated experiments at each DNA concentration were very similar, indicating that the μCA device for dPCR testing achieves high accuracy in a wide dynamic range.

## 3 Discussion

The sustained pursuit of rapid and high‐throughput droplet generation is the focus of new‐generation microfluidics. In this work, we designed an integrated μCA device, which includes a μCA chip and two matching containers for water and oil, respectively. The containers are compatible with syringes or 96‐well plates, which make the device a strong candidate for droplet generation through various modes, with advantages in terms of high‐throughput, portability, and parallelization. Compared to traditional microfluidic systems, our μCA device incorporated parallel channels and nozzles to elevate the droplet generation rate. High‐throughput and parallel droplet production are enabled by a simple yet robust μCA device design, which has great potentials for high‐throughput single‐cell and single‐molecule analysis, and large‐scale synthesis of hydrogel microparticles.^[^ ^28^ ^]^ Moreover, the μCA device will greatly expand the functionalities to meet different application needs across biology and medicine. For example, rapid collection and detection of nucleic acid molecules at high sensitivity and accuracy by dPCR is highly desired for point‐of‐care diagnosis.^[^ ^29^ ^]^ This requires the development of a portable, miniaturized, and low‐cost droplet generation device. The μCA device reported in this work meets these criteria, as its high‐throughput, integrated, and portable features provide a platform for qualitative and quantitative detection of nucleic acid molecules. Furthermore, the μCA device can be massively manufactured by microfabrication facilities with high reproducibility at relatively low material costs. The simplicity and reliability of this method have made droplet generation accessible to many nonexpert labs and commercial entities. It is envisioned that the μCA device coupled with other developing detection and/or reaction methodologies will have a significant impact on both laboratory and industrial applications.

## 4 Conclusion

In summary, we developed a versatile μCA device for high‐throughput generation of monodispersed droplets. A versatile droplet platform was demonstrated by adopting various droplet generation modes to meet various application needs, including mechanical pump‐based large‐scale droplet generation, handheld syringe‐based portable droplet generation, and gas pump‐based highly parallel droplet generation. Digital amplification and quantification of nucleic acid were achieved with high accuracy by utilizing the μCA device for partitioning the target templates into monodispersed droplets. Furthermore, the μCA device can be massively manufactured at relatively low martial costs, offering opportunities for many biomedical laboratories to produce monodispersed droplets with their application needs. We believe that this reliable, miniaturized, and cost‐effective device can find broad applications across the fields of material science, biology, and medicine.

## 5 Experimental Section

5.1

5.1.1

##### Materials and Reagents

Oligo‐DNAs of T790M DNA template, primers, and TaqMan probe were synthesized by Sangon Biotech (Shanghai). AceQ Universal U + Probe Master Mix V2 was purchased from Vazyme Company (Nanjing). Mineral oil with surfactant was provided by Xinhaohui Biotech (Hangzhou). 1H,1H,2H,2H‐perfluorooctyltrichlorosilane (PFOTCS) and isopropyl alcohol (IPA) were purchased from Sigma. Disposable syringes (1 and 3 mL) were purchased from BD Company. 1X phosphate buffer saline (PBS) and ddH_2_O were obtained from Sangon Biotech (Shanghai) and used as received. Other reagents were purchased from Adamas‐beta unless otherwise noted.

##### μCA Fabrication

The fabrication of μCA devices was entrusted to Boyan Company (Suzhou, China). A photomask was first designed using computer‐assisted designs according to our previous work.^[^ ^24^ ^]^ The pattern was then transferred onto silicon wafer through photolithography followed by inductively coupled plasma (ICP) etching: 1) photoresist (AZ514) was spin‐coated onto a 6 inch cleaned silicon wafer (600 rpm for 5 s, 4000 rpm for 30 s), prebaked at 95 °C for 90 s; 2) exposure to UV source (Karl SUSS MA6) with the designed photomask for 6.5 s; 3) rinsing with 2.38% TMAH for 45 s, and postbaking at 100 °C for 2 min; and 4) ICP etching parameters: C4F8 190 sccm, SF6 450 sccm, RF Etch power 50 W, RF Pass power 20 W, ICP Etch power 2300 W, ICP Pass power 1650 W, 6 μm min^−1^. The obtained μCAs were washed with IPA and then permanently bonded with a glass through anodic bonding. On a 6 inch wafer, over 800 μCA were constructed, and laser cutting was finally employed to obtain individual μCA.

##### Microchannel Surface Hydrophobic Treatment

The microchannel surfaces were treated with PFOTCS for hydrophobic coating to produce monodispersed and reliable droplets before implementation. The μCAs were cleaned by immersion in a piranha solution (*i.e.*, H_2_SO_4_/H_2_O_2_ = 3/1, v/v) and ultrasonication for 10 min. After ultrasonication, the μCA were washed with deionized water and dried under 70 °C for 6 h. The cleaned μCA were then put into an oxygen plasma cleaner for surface activation. The activated μCAs were transferred into a vacuum desiccator, which contains an open vial with PFOTCS. Later, the desiccator was vacuumed for 1 h. Subsequently, the μCAs were heated on a hot plate at 120 °C for 5 min. Finally, the μCAs were washed with IPA and then deionized water, and dried in ambient environment.

##### Droplet Generation and Characterization

The μCA device can be operated with three kinds of modes as described: i) for mechanical pump‐based large‐scale droplet generation, the PBS buffer was loaded into a 3 mL syringe, the syringe port was then inserted into the water container. Subsequently, the device was immersed in a beaker containing an oil phase (mineral oil or Novec 7500). Large‐scale monodispersed droplets can be continuously generated in the beaker with controlled flow rates with a mechanical pump (Harvard apparatus); ii) For handheld syringe‐based portable droplet generation, 50 μL of PBS buffer was first loaded into the water container. The inlet of the container was then connected with a 1 mL syringe. Afterward, the μCA was immersed into 150 μL of mineral oil in an oil cup. The pressure generated by the handheld injection squeezes the aqueous phase into the μCA, and forms droplets at the nozzles. The whole process takes less than 30 s; and iii) For air pump‐based highly parallel droplet generation, sample solution was first added to the water container, and then the device was immersed into the oil phase cup. The sample solution was dispersed into the oil phase to form droplets by the gentle pressure generated by the parallel air pump. The oil cups were placed in a 96‐well plate so that up to 96 samples could be emulsified simultaneously with an integrated air pump. Micrographs of the droplets were obtained with ECLIPSE Ti2 microscopy (Nikon). The average diameter and standard deviation of the droplets were evaluated from microscopy images using the Image‐Pro Plus software. For each droplet population, more than 300 objects were measured to calculate the average diameter.

##### dPCR

The μCA device was applied to produce droplets for dPCR. dPCR was performed in a 25 μL reaction, consisting of 12.5 μL 2X Mater Mix (Vazyme), 5 μL ddH_2_O, 2 μL 20 μM primers, 0.5 μL 10 μM TaqMan probe, and 5 μL DNA template (Table S3, Supporting Information). We used a set of two primers and a probe synthesized by Sangon Biotech (Shanghai) to quantify the Epidermal Growth Factor Receptor (EGFR) T790M mutation gene (Table S2, Supporting Information). The standard DNA template was serially diluted in TE buffer to a concentration ranging from ≈10 000 to 1 copies/μL. We employed an integrated air pump to generate droplets with zero dead volume within 3 min. The PCR thermocycling was performed directly with the receptor oil cup on a conventional PCR thermal cycler: 95 °C for 5 min, 45 cycles of 95 °C for 10 s, and 58 °C for 30 s, and finally held at 25 °C. The droplets were imaged using the fluorescent inverted microscope (Ti2‐U, Nikon). Positive and negative droplets counting was processed with the QX200 droplet reader (Bio‐Rad), and the data were analyzed using a professional software (QX Manager Software, Bio‐Rad).

## Conflict of Interest

Y. Wang and Z. Tang have filed a patent related to this work.

## Author Contributions

Z.T. and Y.W. conceived of the idea and designed experiments. Z.T. and C.L. designed the μCA devices, generated droplets, performed PCR, collected images, and analyzed data. Z.T. prepared the manuscript. Y.W., D.E.R., and J.K. revised the manuscript. This work was supervised by Y.W.

## Supporting information

Supplementary Material

## Data Availability

The data that support the findings of this study are available from the corresponding author upon reasonable request.
