# Supplementary material for: A Versatile Microchannel Array Device for Portable and Parallel Droplet Generation
Source: Small Sci. 2024 Mar 21;4(5):2400005. doi: 10.1002/smsc.202400005 (PMC11935260; doi:10.1002/smsc.202400005)
Supplement: Supplementary file 1 — Supplementary Material [file SMSC-4-2400005-s001.zip › smsc.202400005-sup-0001-suppdata-S1.pdf]

Supplementary Information

A Versatile Microchannel Array Device for Portable and Parallel Droplet Generation

*Zhengmin Tang, David Eun Reynolds, Caishu Lv, Dandan Zhang, Jina Ko, and Yongcheng Wang**

Supplementary Tables

Table S1. Material costs estimation of the fabrication of μCA device**.**

**Materials**[a]	**Cost (USD $)**	**Average (USD $)**
6-inch silicon	30	0.0375
Glass	20	0.025
Mask	120	0.15
Photolithography reagent	100	0.125
	Total: ~270	Average: ~ **0.337**

[a]Over 800 μCA chips can be fabricated in a 6-inch silicon water, other consumables (*i.e.*, oil cup, aqueous solution inlet) are made of plastics and the costs can be negligible compared with above materials described.

Table S2. Template DNA, primer and probe used in dPCR analysis**.**

**Template DNA**	**Primer/probe 5'-3'**
T790M	FP: GCCTGCTGGGCATCTG<br>RP: TCTTTGTGTTCCCGGACATAGTC<br>Probe: Fam-ATGAGCTGCATGATGAG-MGB

Table S3. Recipe of 2X master mix for dPCR in this work**.**

For 25 µL premix	Vol. (µL)	Stock conc.	Final conc.
Polymerase master mix	12.5	2X	1X
Forward primer	1	20 µM	800 nM
Reverse primer	1	20 µM	800 nM
TaqMan probe	0.5	10 µM	200 nM
Template DNA	5	--	--
ddH_2O	5	--	--

Supplementary Figures

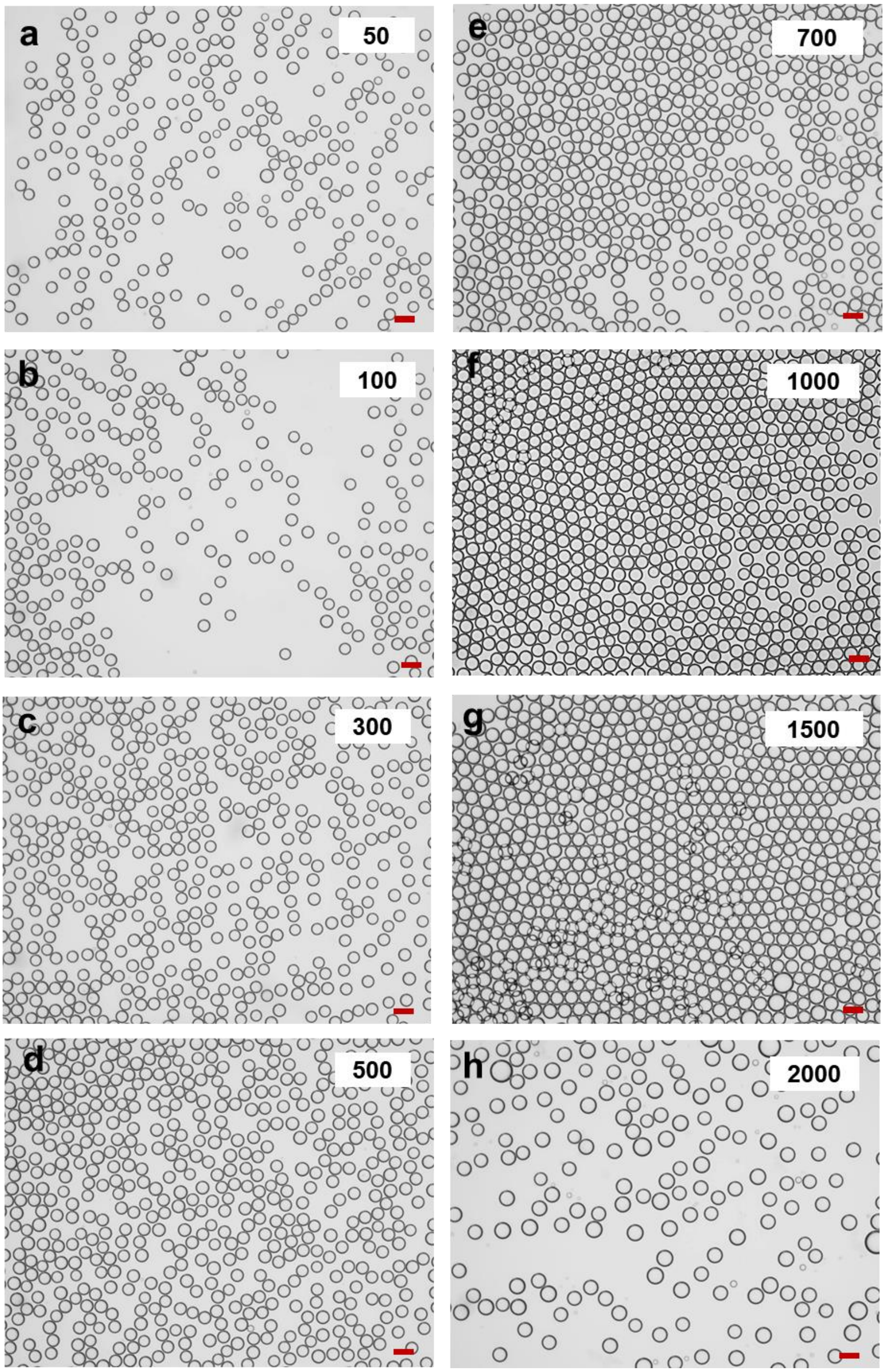

Figure S1. Microscopy images of the size and morphology of droplets generated under varied flow rate (μL/h) by syringe pump injection. Scale bars: 100 μm.

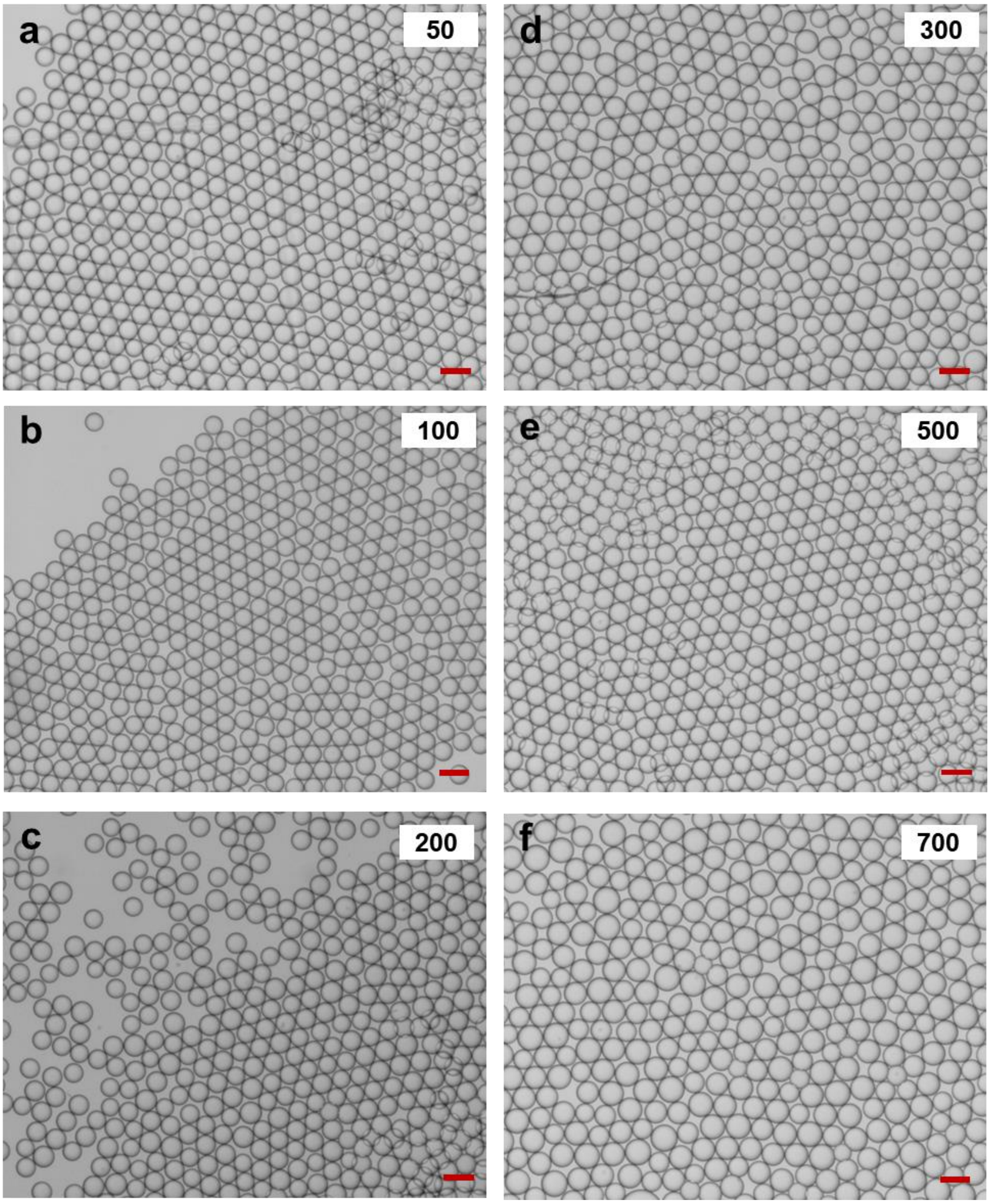

Figure S2. Microscopy images of the size and morphology of droplets generated under varied flow rate (μL/h) by syringe pump injection. Novec 7500 oil was used as the continuous phase. Scale bars: 100 μm.

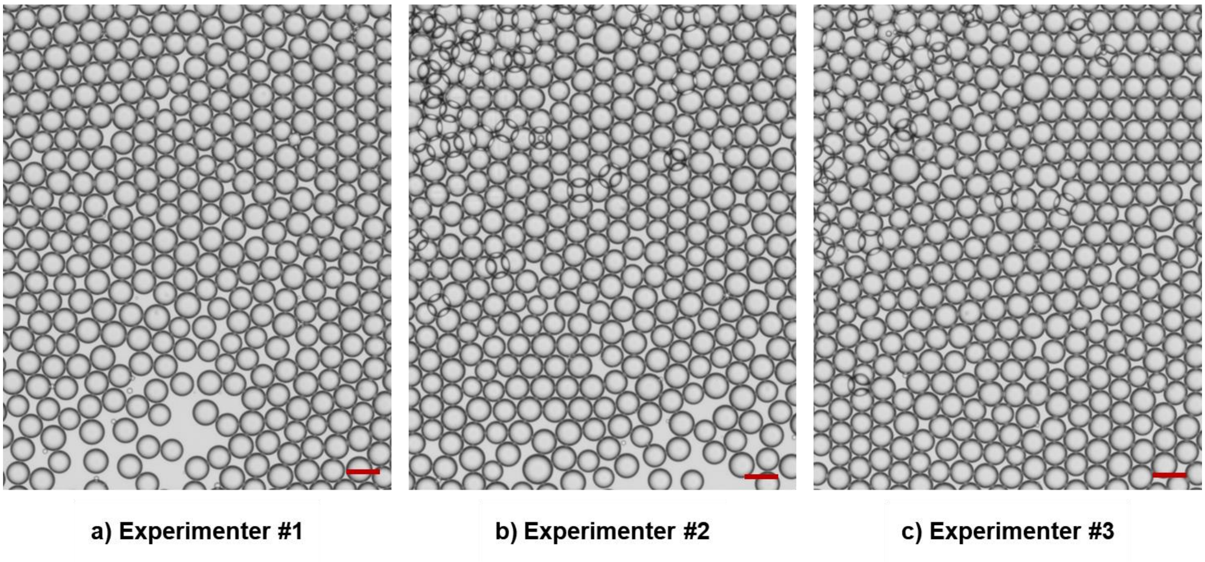

Figure S3. Microscopy images of droplets generated by handheld syringe injection by three different experimenters. Scale bars: 100 µm.
